# Supplementary material for: Quantum interference directed chiral raman scattering in two-dimensional enantiomers
Source: Nat Commun. 2022 Mar 10;13:1254. doi: 10.1038/s41467-022-28877-6 (PMC8913836; doi:10.1038/s41467-022-28877-6)
Supplement: Supplementary file 1 — Supplementary Information [file 41467_2022_28877_MOESM1_ESM.pdf]

Supplementary Materials to

"Quantum Interference Directed Chiral Raman Scattering in Two-dimensional Enantiomers"

Shishu Zhang,[†,#] Jianqi Huang,[‡,§,#] Yue Yu,[†] Shanshan Wang,[†] Teng Yang,*[,‡,§] Zhidong Zhang,[‡,§†] Lianming Tong*[,†] and Jin Zhang[†]

[†] Center for Nanochemistry, Beijing Science and Engineering Center for Nanocarbons, Beijing National Laboratory for Molecular Sciences, College of Chemistry and Molecular Engineering, Peking University, 100871, Beijing, China

[‡]Shenyang National Laboratory for Materials Science, Institute of Metal Research, Chinese Academy of Sciences, 72 Wenhua Road, Shenyang 110016, China

[§]School of Material Science and Engineering, University of Science and Technology of China, Shenyang 110016, China.

*Corresponding authors: TY (yangteng@imr.ac.cn); LT (tonglm@pku.edu.cn)

[#] These authors contributed equally

1. Crystal structure of ReS_2

2. The optical images and AFM characterizations of 1L ReS_2 and $ReSe_2$ flakes

3. Electronic structure and phonon dispersion curve of 1L ReS_2

4. Quantum interference in ReS_2

5. The polar plots of chiral responses in 1L ReS_2 and 1L $ReSe_2$ flakes

1. Crystal structure of ReS_2

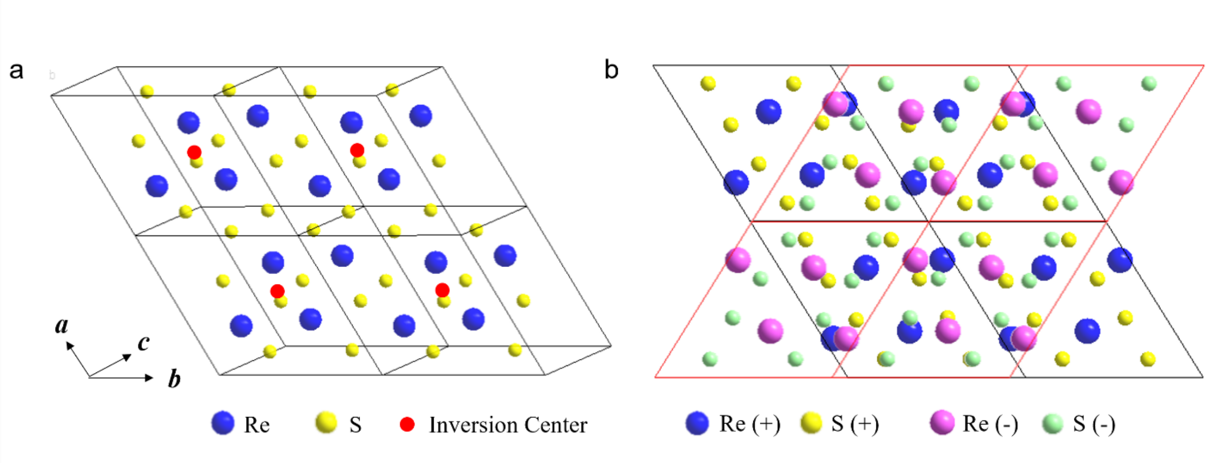

Supplementary Figure 1. Schematics of three dimensional (a) and two dimensional (b) ReS_2 structure.

2. The optical images and AFM characterizations of 1L ReS_2 and 1L $ReSe_2$ flakes

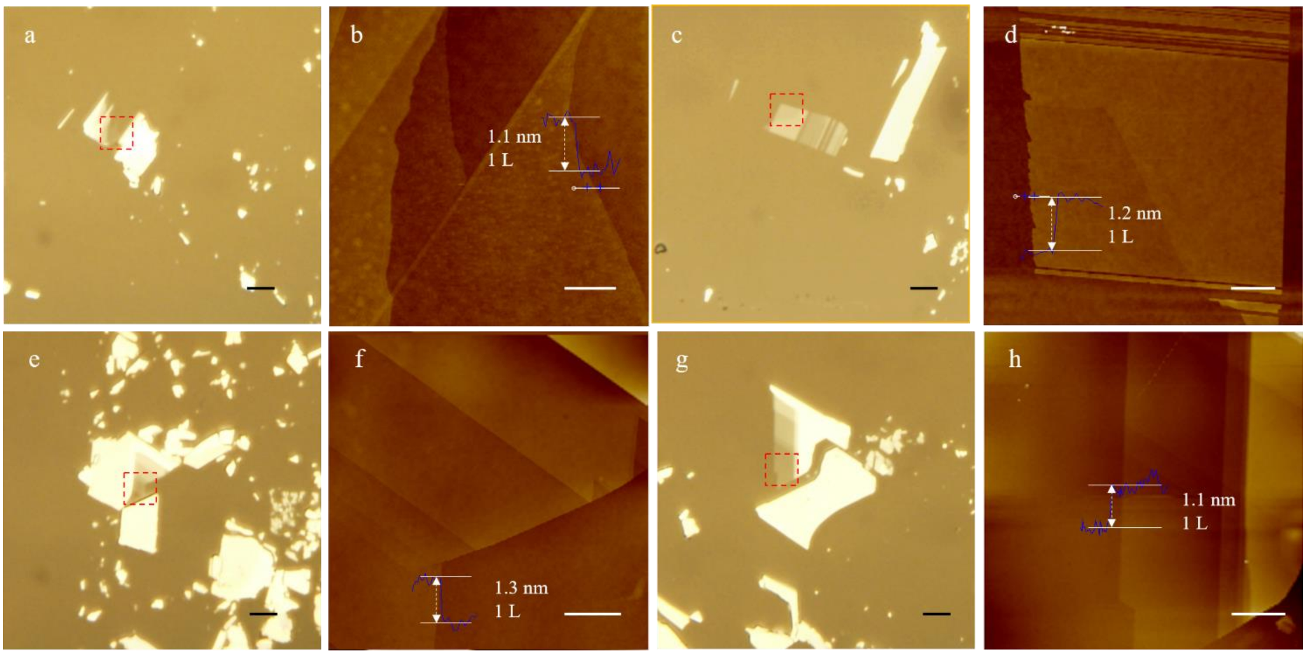

Supplementary Figure 2. The optical and AFM characterizations of 1L ReS_2 (a-d) and 1L $ReSe_2$ (e-h). The scale bars in the optical image are 5 μm, and in AFM images are 1μm.

The mechanical exfoliated ReS_2 and $ReSe_2$ were characterized and located by optical microscope. The layers of flakes were determined by atomic force microscope. The AFM images shown in Supplementary Figure 2 reveal the average thicknesses of the mono-layer ReX_2 are 1.1 nm ~ 1.3 nm, which are slightly larger than the predicted (about 0.7 nm). The discrepancies are owing to the offsets in AFM instrument[1].

3. Electronic structure and phonon dispersion relation of 1L ReS_2

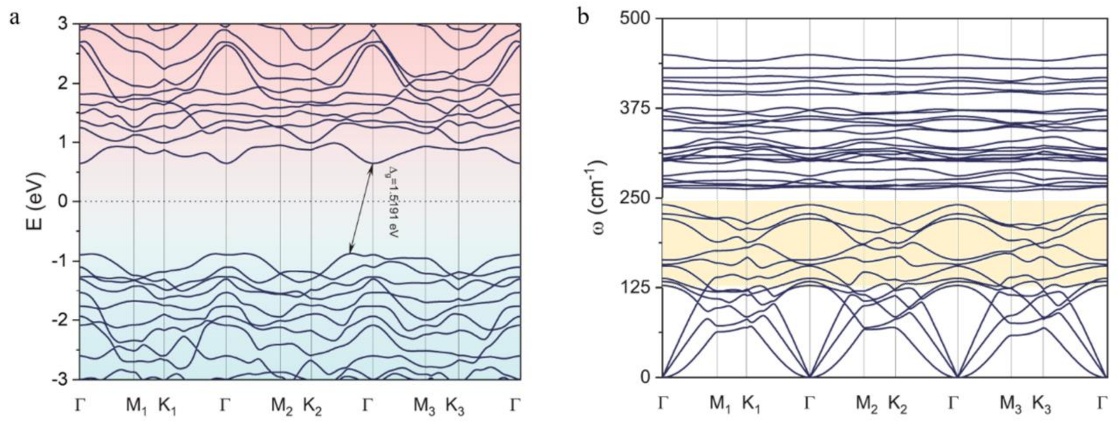

Supplementary Figure 3. Calculated electronic band structure (a) and phonon dispersion relation (b) of 1L ReS_2.

4. Quantum interference in ReS_2

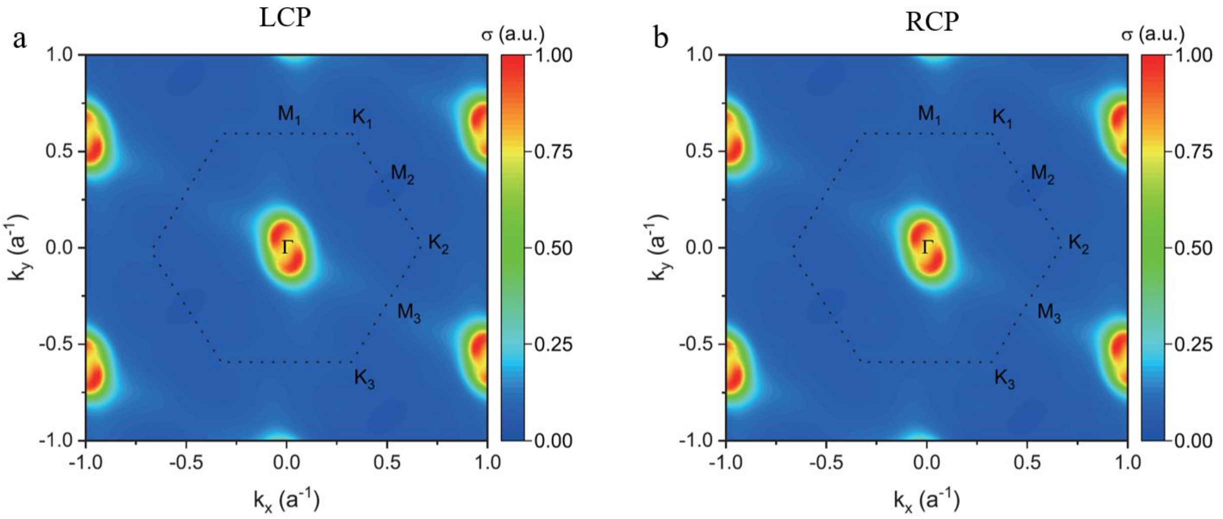

Supplementary Figure 4. Optical absorption of (a) left circularly polarized and (b) right circularly polarized laser of 1.96 eV.

In this section, we aim to reveal the microscopic picture behind the interference effect in chiral Raman scattering in 1L ReS_2. First of all, we look into the optical absorption and define optical absorption cross section $\lambda \sim \sum_{\mathbf{k},f,i} |\mathbf{D_{fi}}(\mathbf{k}) \cdot \mathbf{e_i}|^2$, in which $\mathbf{D_{fi}}(\mathbf{k})$ and $\mathbf{e_i}$ are the dipole vector due to vertical electronic photoexcitation from i to f state and polarization vector of incident laser, respectively. $\mathbf{D} = (D_x, D_y) = (d_x e^{i\alpha}, d_y e^{i\beta})$, and $\mathbf{e_i}$ can be left circular polarization LCP ($\boldsymbol{\sigma}_+ = (1, i)$) or right circular polarization RCP ($\boldsymbol{\sigma}_- = (1, -i)$). The calculated optical absorption excited by both RCP and LCP are found to be identical, as shown in Supplementary Figure 4. To satisfy the condition of helicity-independent optical absorption or $|\mathbf{D_{fi}}(\mathbf{k}) \cdot \boldsymbol{\sigma}_+|^2 = |\mathbf{D_{fi}}(\mathbf{k}) \cdot \boldsymbol{\sigma}_-|^2$, it should have $\alpha - \beta = n\pi$, n is an integer number. This is indeed the case, as seen from Supplementary Figure 4 (e, f). Supplementary Figure 4 (e) and (f) show respectively D_x and D_y (for better visualization, D_y is rotated counterclockwise by 90 degrees) in complex plane and the phase difference $\alpha - \beta$ between D_x and D_y for a single photon excitation process from the top valence band to the bottom conduction band at each k point. Obviously the D_x and D_y of $\mathbf{D}$ for each single photoexcitation process are either parallel or antiparallel, contributing to the helicity-independence of optical absorption.

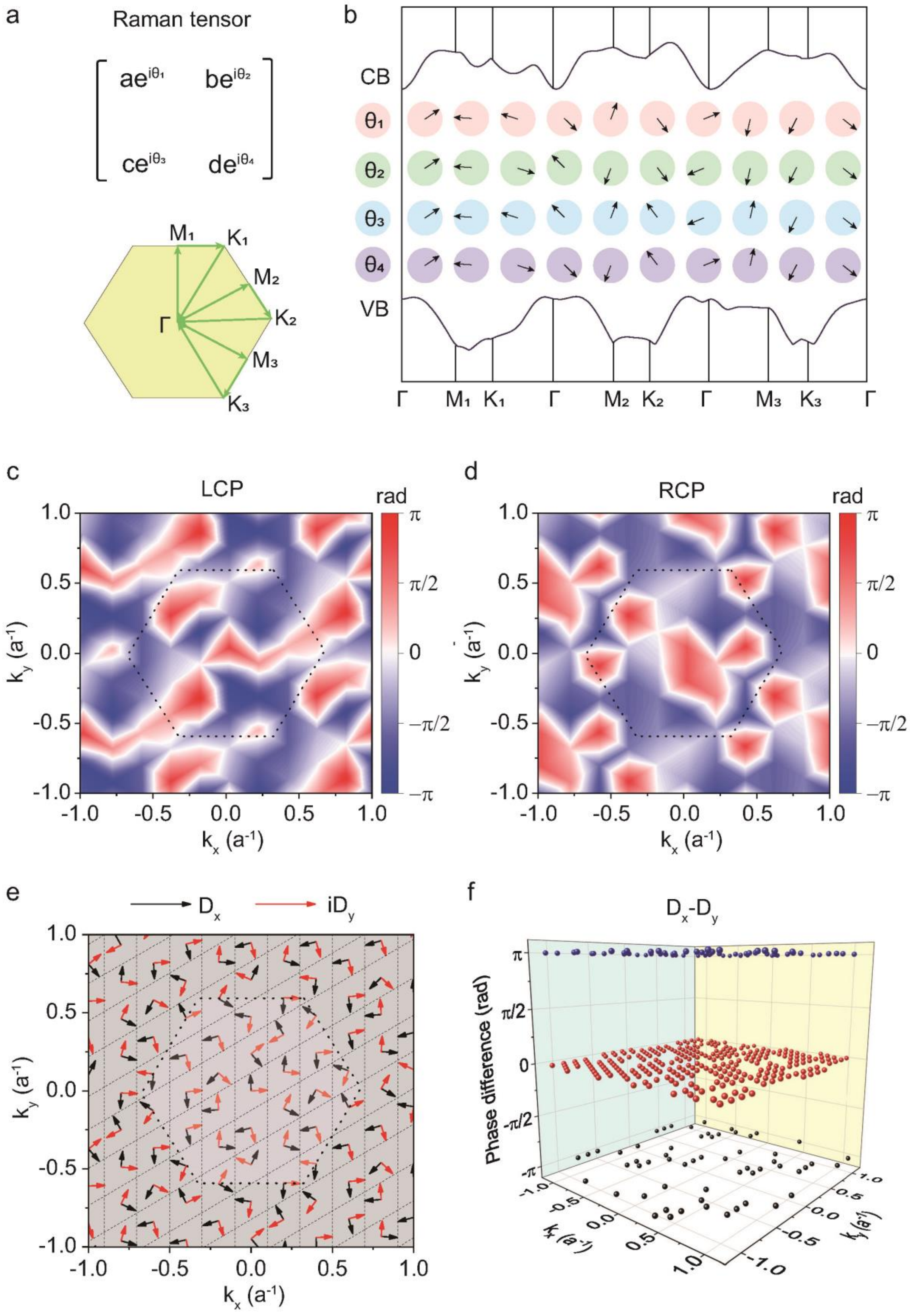

Supplementary Figure 5. (a) Generalized Raman tensor with different phases for each component. (b) Visualization of the 4 phases in Raman tensor between the typical top valence and bottom conduction bands. E_L = 1.96 eV and mode

IV is chosen. (c, d) **k**-resolved phase of $\mathbf{T_{i,f,n,n'}(k)} = \mathbf{e_s} \cdot \mathbf{R_{i,f,n,n'}(k)} \cdot \mathbf{e_i}$ for (c) left circular polarized and (d) right circular polarized lasers. (e) **k**-resolved dipole vector **D** = (D_x, D_y) between the two bands in (b). The y-component is rotated counter-clockwise by 90 degrees. (f) Phase difference between x- and y-component of dipole vector **D** in each **k**-point.

Then we look into resonant Raman scattering process. Let us still focus on one electron Raman scattering process between two single electronic bands. We define a generalized Raman tensor in Supplementary Figure 5(a), each term has an independent phase angle (θ_1, θ_2, θ_3, θ_4). Raman intensity can be evaluated by the following,

$$I_{i,f,n,n'} \propto \int_0^{2\pi} \left| (\cos\beta \quad \sin\beta) \begin{pmatrix} a e^{i\theta_1} & b e^{i\theta_2} \\ c e^{i\theta_3} & d e^{i\theta_4} \end{pmatrix} \begin{pmatrix} 1 \\ \pm i \end{pmatrix} \right|^2 d\beta$$

$$= \pi(a^2 + b^2 + c^2 + d^2) \pm 2\pi[ab\sin(\theta_1 - \theta_2) + cd\sin(\theta_3 - \theta_4)] \qquad \text{Eq. (S1)}$$

In Supplementary Figure 5(b) we show the calculated phase angles between two single bands without any inter-**k** interference effect considered. All the four phase angles are either parallel or antiparallel to each other, giving rise to Raman intensity independent on helicity, as seen from Eq. (S1). The phase difference between the components of Raman tensor (θ_i - $\theta_j = n\pi$, in which i, j = 1, 2, 3, 4, $i \neq j$, and n is an integer number) at each **k**-point can be deduced from the relationship between D_x and D_y. To reveal the interference origin behind the chiral Raman scattering, we show the **k**-resolved phase of complex $\mathbf{T_{i,f,n,n'}(k)} = \mathbf{e_s} \cdot \mathbf{R_{i,f,n,n'}(k)} \cdot \mathbf{e_i}$ in Supplementary Figure 5 (c, d). Very different phase distributions are found between LCP and RCP, leading to different interference between **k**-points, and ultimately distinctive intensities. Both constructive and destructive interference between **k**-points finally give rise to vastly different Raman tensors for the 6 Raman modes, as indicated in Supplementary Table 1 and Supplementary Table 2 with considering the symmetry of ReS_2.

Mode I	Mode II	Mode III
$\begin{pmatrix} 0.22e^{0.98i} & 0.37e^{-2.52i} \\ 0.37e^{-2.52i} & 1.00e^{2.26i} \end{pmatrix}$	$\begin{pmatrix} 1.03e^{-1.88i} & 0.43e^{-2.49i} \\ 0.43e^{-2.49i} & 0.91e^{2.29i} \end{pmatrix}$	$\begin{pmatrix} 0.43e^{-1.98i} & 0.10e^{1.97i} \\ 0.10e^{1.97i} & 1.00e^{-1.26} \end{pmatrix}$
Mode IV	Mode V	Mode VI
$\begin{pmatrix} 1.00e^{0.61i} & 0.16e^{1.84i} \\ 0.16e^{1.84i} & 0.72e^{-0.24i} \end{pmatrix}$	$\begin{pmatrix} 0.35e^{2.11i} & 0.98e^{0.09i} \\ 0.98e^{0.09i} & 0.97e^{-0.79i} \end{pmatrix}$	$\begin{pmatrix} 1.00e^{0.56i} & 0.37e^{1.58i} \\ 0.37e^{1.58i} & 0.55e^{1.44i} \end{pmatrix}$

Supplementary Table 1. Raman tensor element of different Raman vibrational modes excited by 2.33 eV photon energy.

Mode I	Mode II	Mode III
$\begin{pmatrix} 0.94e^{-0.07i} & 0.60e^{-0.21i} \\ 0.60e^{-0.21i} & 1.00e^{-2.26i} \end{pmatrix}$	$\begin{pmatrix} 1.00e^{0.14i} & 0.13e^{-2.91i} \\ 0.13e^{-2.91i} & 0.53e^{-2.40i} \end{pmatrix}$	$\begin{pmatrix} 0.5325e^{2.17i} & 0.71e^{0.92i} \\ 0.71e^{0.92i} & 1.00e^{1.16i} \end{pmatrix}$
Mode IV	Mode V	Mode VI
$\begin{pmatrix} 0.94e^{-3.07i} & 0.36e^{0.10i} \\ 0.36e^{0.10i} & 0.69e^{1.26i} \end{pmatrix}$	$\begin{pmatrix} 0.30e^{-2.66i} & 0.42e^{-2.08i} \\ 0.42e^{-2.08i} & 1.00e^{-2.23i} \end{pmatrix}$	$\begin{pmatrix} 0.32e^{-2.54i} & 0.49e^{-2.39i} \\ 0.49e^{-2.39i} & 1.00e^{-2.36i} \end{pmatrix}$

Supplementary Table 2. Raman tensor element of different Raman vibrational modes excited by 1.96 eV photon energy.

5. The polar plots of chiral response in 1L ReS_2

The polarization states of the incident light change from linearly, elliptically to circularly polarized. The Raman intensities variation with rotation angle θ can be fitted by:

$$I \propto \frac{1}{2}\left|\begin{pmatrix} ae^{i\varphi_a} & be^{i\varphi_b} \\ be^{i\varphi_b} & ce^{i\varphi_c} \end{pmatrix} \cdot \begin{pmatrix} 1 - i\cos 2\theta & -i\sin 2\theta \\ -i\sin 2\theta & 1 + \cos 2\theta \end{pmatrix} \cdot \begin{pmatrix} 1 \\ 0 \end{pmatrix}\right|^2$$

$$I = \frac{1}{2}[a^2 + a^2\cos^2 2\theta + 2b^2 + c^2\sin 2\theta + 2ab\sin 2\theta\sin(\varphi_b - \varphi_a) + 2bc\sin 2\theta\sin(\varphi_c - \varphi_b)$$
$$+ ab\sin 4\theta\cos(\varphi_a - \varphi_b) + bc\sin 4\theta\cos(\varphi_c - \varphi_b)]$$

For ReS_2 (-), $be^{i\varphi_b}$ should be replaced by $-be^{i\varphi_b}$.

The Raman intensities of RCP (I_R) for 132 cm^{-1} and 143 cm^{-1} modes are higher in ReS_2 (+) than that in ReS_2 (-), and the intensity of 235 cm^{-1} is higher with LCP in ReS_2 (+). The results of ReS_2 (-) are opposite to ReS_2 (+).

2.33 eV	Mode I (~132 cm^{-1})	Mode II (~143 cm^{-1})	Mode III (~153 cm^{-1})	Mode IV (~162 cm^{-1})	Mode V (~212 cm^{-1})	Mode VI (~235 cm^{-1})
1 L ReS_2 (+)	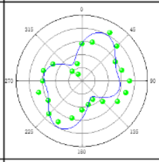	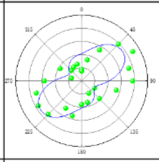	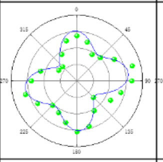	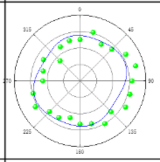	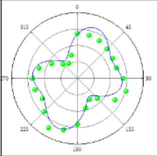	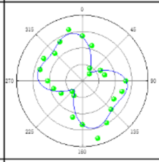
1 L ReS_2 (-)	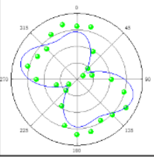	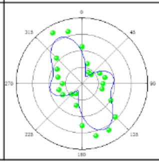	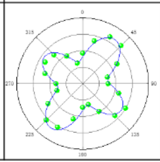	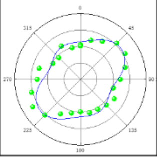	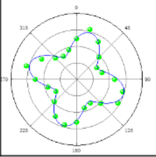	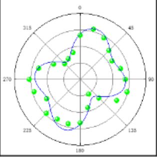

Supplementary Table 3. Raman intensities of 1L ReS_2 with different rotation angles of QWP. The excitation photon energy is 2.33 eV.

1.96 eV	Mode I (~132 cm^{-1})	Mode II (~143 cm^{-1})	Mode III (~153 cm^{-1})	Mode IV (~162 cm^{-1})	Mode V (~212 cm^{-1})	Mode VI (~235 cm^{-1})
1 L ReS_2 (+)	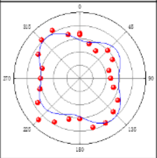	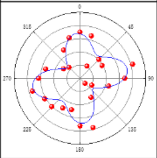	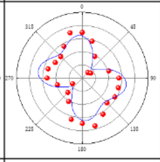	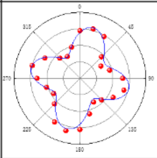	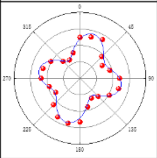	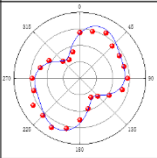
1 L ReS_2 (-)	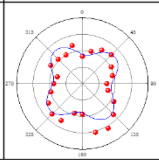	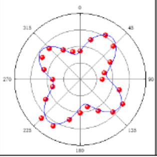	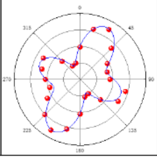	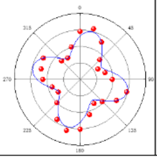	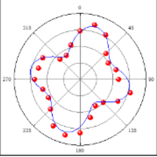	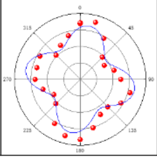

Supplementary Table 4. Raman intensities of 1L ReS_2 with different rotation angles of QWP. The excitation photon energy is 1.96 eV.

2.33 eV	~110 cm^{-1}	~119 cm^{-1}	~125 cm^{-1}	~162 cm^{-1}
1 L $ReSe_2$ (+)	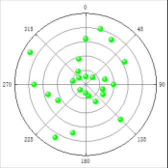	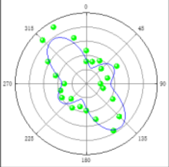	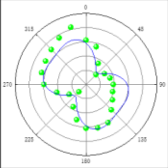	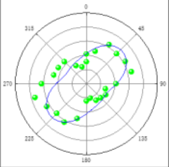
1 L $ReSe_2$ (-)	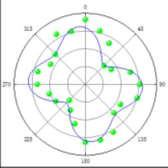	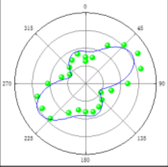	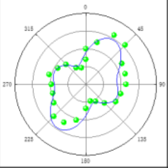	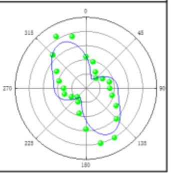

Supplementary Table 5. Raman intensities of 1L $ReSe_2$ with different rotation angles of QWP. The excitation photon energy is 2.33 eV.

1.96 eV	~110 cm^{-1}	~119 cm^{-1}	~125 cm^{-1}	~162 cm^{-1}
1 L $ReSe_2$ (+)	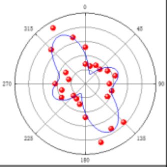	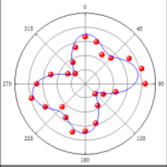	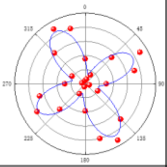	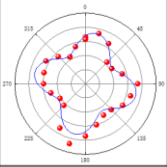
1 L $ReSe_2$ (-)	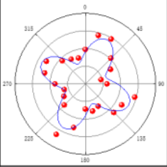	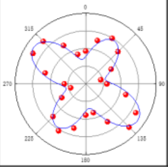	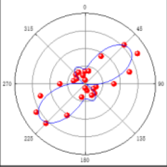	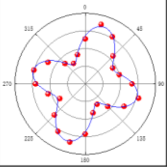

Supplementary Table 6. Raman intensities of 1L $ReSe_2$ with different rotation angles of QWP. The excitation photon energy is 1.96 eV.

References

1. Nemes-Incze, P., Osváth, Z., Kamarás, K. & Biró, L. P. Anomalies in thickness measurements of graphene and few layer graphite crystals by tapping mode atomic force microscopy. *Carbon* **46**, 1435-1442 (2008).
